# Management of laryngomalacia: experience with 22 cases

**DOI:** 10.1016/S1808-8694(15)31331-8

**Published:** 2015-10-20

**Authors:** Melissa A.G. Avelino, Raquel Y.G. Liriano, Reginaldo Fujita, Shirley Pignatari, Luc L.M. Weckx

**Affiliations:** ^1^ M^1^ Physician, Ph.D. studies under course, Discipline of Pediatric Otorhinolaryngology, Federal University of Sao Paulo – Escola Paulista de Medicina (UNIFESP-EPM); ^2^ Physician, Specialization under course, Discipline of Pediatric Otorhinolaryngology, Federal University of Sao Paulo – Escola Paulista de Medicina (UNIFESP-EPM); ^3^ Professor, Ph.D. and Assistant, Discipline of Pediatric Otorhinolaryngology, Federal University of Sao Paulo – Escola Paulista de Medicina (UNIFESP-EPM); ^4^ Joint Professor and Head of the Discipline of Pediatric Otorhinolaryngology, Federal University of Sao Paulo – Escola Paulista de Medicina (UNIFESP-EPM); ^5^ Full Professor, Head of the Department of Otorhinolaryngology, Federal University of Sao Paulo – Escola Paulista de Medicina (UNIFESP-EPM); Study carried out at Discipline of Pediatric Otorhinolaryngology, Federal University of Sao Paulo – Escola Paulista de Medicina (UNIFESP-EPM)

**Keywords:** laryngomalacia, supraglottoplasty, complications, polysomnography

## Abstract

**L**aryngomalacia is the most frequent cause of stridor in childhood, and in most of the cases, spontaneous resolution occurs by the age of 2 years. Approximately 10% of the cases (severe laryngomalacia) require surgery. This condition is of unknown etiology and its diagnosis is made by fiberoptic laryngoscopy, which shows shortening of the aryepiglottic folds, and/or redundant arytenoid mucosa, and/or anterior-posterior epiglottic prolapse. **Aim**: Our objective was to verify the main clinical and anatomical affections and to highlight the clinical parameters for clinical follow-up and surgical indication in patients with laryngomalacia. **Study design**: Transversal cohort study. **Material and Method**: Twenty-two children diagnosed with laryngomalacia in the Pediatric Otorhinolaryngology of UNIFESP-EPM, from January 2001 to December 2003, whose clinical and surgical follow-up were performed by the same examiner, were enrolled in this study. **Results**: Out of twenty-two evaluated children, 2 (9.1%) presented with severe laryngomalacia and pectus excavatum (funnel chest). At polysomnography, no child presented any significant respiratory event during sleeping. Those two children with severe laryngomalacia were submitted to supraglottoplasty with resection of the aryepiglottic folds. **Conclusion**: We concluded that stridor and shortening of the aryepiglottic folds are preponderant in children with laryngomalacia. The polysomnographic exam did not prove to be a good parameter for clinical follow-up, neither for surgical indication. The most important parameters were pectus excavatum and failure to thrive. Supraglottoplasty is effective and has low morbidity rate.

## INTRODUCTION

Laryngomalacia is the most common cause of stridor in childhood years, representing 60% to 75% of all cases of laryngeal congenital anomalies1., 2.. It is characterized by the presence of inspiration stridor that is detected within the first 2 weeks of life and normally disappears up to the age of 2 years^3^. About 90% of the cases progress well and do not require treatment and only 10% of the cases (severe laryngomalacia) require surgical intervention. The diagnosis may be defined by videonasopharyngolaryngoscopy in which we can observed supraglottic anatomical affection, with collapse during inspiration. Anatomical affection occurs owing to shortening of aryepiglottic folds, excessive arytenoid mucosa, and anterior-posterior epiglottis drop2., 4..

Etiology is still unknown, but most of the authors believe that the cause is lack of coordination and/or neuromuscular immaturity; more recently, gastroesophageal reflux has been considered also as a cause factor2., 5., 6..

Laryngomalacia may be associated with other upper airways anomalies; thus, some authors recommend the conduction of direct laryngoscopy and routine bronchoscopy for the diagnosis7., 8..

Even though laryngomalacia is the main cause of congenital stridor in childhood^9^, many Otorhinolaryngologists have questioned the clinical and/or surgical treatment of these patients as well as their diagnoses. There are no consensus or very clear parameters in the literature about the exams required for the diagnosis (flexible videonasofibroscopy and/or rigid bronchoscopy) and clinical and/or surgical treatment.

## OBJECTIVE

To demonstrate, based on the experience of our service of Pediatric Otorhinolaryngology, the main clinical and anatomical affections found in patients with laryngomalacia without other congenital or neurological associated affections, as well as to detect which are the important parameters for clinical follow up and surgical indication.

## MATERIAL AND METHOD

The study included children with diagnosis of laryngomalacia without other associated congenital anomalies seen at the ambulatory of Pediatric Otorhinolaryngology, Federal University of Sao Paulo - Escola Paulista de Medicina, between January 2001 and December 2003. The ambulatory in our service sees cases of pediatric laryngology already screened by the institution. Since 2001, we have followed a protocol approved by the ethics and research committee of the institution in which children with diagnosis of respiratory stridor are submitted to detailed anamnesis, videonasofibroscopy polysomnography and, if necessary, rigid bronchoscopy. In the anamnesis, mothers are asked about the onset of stridor, worsening and improvement factors, perinatal history, presence of other congenital anomalies, difficulties during breastfeeding, weight gain, cyanosis, apnea and any affections that might have required hospitalization owing to upper airway obstruction. Our subjects were submitted to nasopharyngolaryngoscopy using 3.2mm diameter flexible fiber in outpatient setting. We did not perform introduction of the flexible endoscope through the vocal folds; when the procedure was required, we used the operating room under sedation and spontaneous ventilation.

In this study we included only cases of laryngomalacia. All other causes of stridor such as subglottic stenosis, vocal fold paralysis, among other forms, were excluded, as well as the cases of laryngomalacia associated with other congenital and/or neurological abnormalities. By assessing image recordings (cassette tapes) of the cases, we divided laryngeal anomalies found in the supraglottis into 3 different types: type I (redundancy of arytenoid mucosa), type II (shortening of aryepiglottic folds), and type III (drop in epiglottis in the anterior-posterior direction), and there were some patients with more than one type of affection.

After the diagnosis of laryngomalacia, all patients were submitted to polysomnography, and then we started the administration of bromopride at the dose of 0.5 to 1mg/Kg/day.

Moreover, stricter pediatric follow up was required concerning weight gain and mothers were informed about possible events. A new assessment was carried out every 2 months and earlier, if necessary. Direct laryngoscopy and bronchoscopy in the operating room were performed only in patients that did not present good clinical evolution, to exclude concomitant lesions. In patients submitted to surgical treatment (supraglottoplasty), we conducted as a routine rigid endoscopy with endoscope 2.7 or 4mm and 0°. In cases of severe laryngomalacia, the initially advocated surgical treatment was sectioning of bilateral aryepiglottic folds.

## RESULTS

We included in the study 22 patients with diagnosis of laryngomalacia, 11 male and 11 female subjects. The age at initial diagnosis ranged from 3 days to 6 months. All of them were followed up every 2 months up to complete remission of stridor. All children (100%) presented inspiration stridor that worsened with effort and retraction of furculum during stridor. Out of the total, 14 (63.3%) presented stridor at birth, and 8 (36.4%) presented stridor for the first time between the first and the second week after birth. Nine patients (40.9%) had difficulties during breastfeeding, manifesting mainly choking. Eight patients (36.4%) presented gastroesophageal reflux diagnosed with two-channel 24-hour ph-meter test. Five patients (22.7%) presented history of episodes of cyanosis during effort (crying, breastfeeding). Two patients (9.1%) progressed with thoracic depression (pectus excavatum) during clinical follow up.

At videonasofibroscopy, all patients (100%) presented epiglottis in omega and type II affections and 12 patients (54.5%) presented associated types I and II. It was not possible to visualize the vocal folds in 5 patients (22.7%) during videonasopharyngolaryngoscopy. Polysomnographic assessment showed that 20 patients (90.9%) did not present sleep obstructive apnea episodes. Some presented mild and transient desaturation of hemoglobulin during active sleep, but it did not characterize apnea. Out of the total 22 children, 15 (68.2%) presented central apnea, with over 20 events, but they were considered compatible with normal range for the age. Two patients (9.1%) presented 1 and 2 events of sleep obstructive apnea episodes not associated with bradycardia and minimum O^2^ saturation (nadir) of 85%.

During follow up, 2 patients (9.1%) did not present good clinical evolution, requiring surgical treatment; they were submitted to supraglottoplasty with aryepiglotic fold section. These two patients aged 2 and 3 months, one male and another female, presented pectus excavatum and dissatisfactory weight gain. Polysomnography did not evidence obstructive apnea in any of the cases. One patients presented 18 events of central apnea without significant desaturation and/or bradycardia; the other presented normal polysomnography, but with desaturation of oxyhemoglobin for approximately 80% upon effort. Videonasofibroscopy did not allow visualization of vocal folds because there was marked collapse of supraglottis; one of them presented type II laryngeal affection, the other had associated types I and II.

## DISCUSSION

In our study, there was no predominance of gender, even though some authors reported predominance of male patients2., 10.. The clinical picture of inspiration stridor found in all our patients with furculum retraction intensified by efforts, is considered the most characteristic by all authors, as well as stridor that may be present since birth or manifest in the first weeks of life.

Nine patients (40.9%) presented difficulty during breastfeeding, especially choking, frequent symptomatology in patients with laryngomalacia that may cause interruption of breastfeeding. The mother should be properly instructed concerning gradual and spontaneous improvement of the symptom, provided that this difficulty is not interfering with weight gain; weight and height deficit, thus, is a decisive factor for supraglottoplasty indication in cases of severe laryngomalacia.

We decided to use a pro-kinetic agent (bromopride) as a routine, because many studies11., 12., 13., 14., 15., 16., 17. have already demonstrated the association between laryngomalacia and gastroesophageal reflux. Bibi^18^ and Yellon^19^ found this association in 70% and 75% of the cases, respectively. Koufman^13^ demonstrated in a study conducted with two-electrode ph-meter test an incidence of 100% of reflux in the studied cases.

To assess the type of laryngeal affection, we conducted as a routine only videonasopharyngolaryngoscopy. In cases submitted to supraglottoplasty, we conducted direct laryngoscopy and rigid bronchoscopy to exclude the presence of syndromic lesions. We agree with some authors2., 12., 20. that consider unnecessary to perform direct laryngoscopy and bronchoscopy as a routine to diagnose laryngeal stridor in children. Gray^21^ demonstrated that in 80% of the cases of children with laryngeal stridor it was possible to make the diagnosis only with flexible endoscopy and in 20% of the cases it was necessary to have direct laryngoscopy with rigid endoscopy. In laryngomalacia, because it is a disease with spontaneous resolution in 90% of the cases, we do not consider it necessary to perform direct laryngoscopy with bronchoscopy as a routine. Even knowing that there may be associated syndromic lesions, we reserve direct laryngoscopy with rigid bronchoscopy for cases that do not have good evolution.

Videonasopharyngolaryngoscopy allows good assessment of the supraglottis and sometime of the glottis as well, but in some cases of laryngolamacia it is not possible to visualize the vocal folds. Even though the literature does not properly define the level of laryngeal affection with severity of laryngomalacia, in both cases submitted to surgical treatment, supraglottic collapse was so marked that it did not allow visualization of vocal folds. The study by Zalzal^22^, in 1987, advocated surgery in those patients in which it was not possible to visualize the vocal folds during supraglottic collapse. We agree that lack of visualization of vocal folds during the exam should serve as a warning signal for the Otorhinolaryngologist, because it may indicate the presence of severe laryngomalacia; however, we do not consider this isolated fact as indication of supraglottoplasty. Despite the fact that 5 of our patients presented this type of laryngeal affection, only two (40%) required supraglottoplasty.

We did not define any correlation between type of laryngomalacia and severity.

Laryngomalacia is considered severe when the child presents symptoms such as cyanosis during breastfeeding, dissatisfactory weight gain, hypoxia and apnea, pectus excavatum, pulmonary hypertension, cor pulmonale or events that require hospitalization owing to upper airway obstruction.

In our sample, we observed that polysomnography did not influence surgical indication; our patients did not present obstructive apnea as expected. In some patients, we observed mild and transient desaturation of oxyhemoglobin during active sleep, but without characterizing obstructive apnea. Two patients (9.1%) presented 1 and 2 events of obstructive apnea, but minimum saturation of O_2_ (Nadir) was 85%; these two children progressed well and did not require supraglottoplasty. The two children submitted to supraglottoplasty did not present obstructive apnea, one of them presented 18 episodes of central apnea and the other had normal polysomnography, even though she had oxyhemoglobin desaturation of approximately 80% upon effort. Even though we detected central type apnea in 15 children (68%) of the 22, it is considered compatible with the age range, owing to neurological immaturity. Thus, the presence of these events of central apnea could reinforce the hypothesis of neuromuscular immaturity as an etiology of laryngomalacia.

The study with polysomnography was not an important factor for the confirmation of severe laryngomalacia, maybe owing to the fact that during sleep, it is not the best moment to detect desaturation of oxyhemoglobin, considering that inspiration stridor is intensified by effort and improved at rest.

The relevant parameters were presence of pectus excavatum and dissatisfactory weight gain. Even though children had had weight gain within the normal range according to the pediatrician, some progressed with significant thoracic depression.

There has been no standardization of parameters to be used for indication of supraglottoplasty so far in the literature. According to most authors, it should be performed in cases of severe laryngomalacia.

In our case study, 9.1% of the patients required surgical treatment because they had severe laryngomalacia, which is in agreement with the literature that reports an incidence that ranges from 10 to 15%.

The technique employed - section of aryepiglottic folds, was described for the first time by Hasslinger^6^, and later it was disseminated by many different authors16., 23., 24.. Lane^10^, in 1984, described the use of micro-scissors to section the aryepiglottic folds as a treatment option for pectus excavatum. Loke^11^, in 2001, reports that the section of aryepiglottic folds in cases of severe laryngomalacia is a quick and effective procedure with very few complications. Thus, it is recommended as the first option, reserving the other types of more extensive procedures for cases of failure in the first procedure.

However, there is no consensus in the literature about the best type of supraglottoplasty. Some authors suggest unilateral section of aryepiglottic fold17., 23., 24., 25. with good results, but about 15% of the patients still require contralateral procedure.

In two patients submitted to bilateral section of aryepiglottic folds, we observed improvement of symptoms within the first 48 hours after surgery, without any type of complications.

## CONCLUSION

We could conclude based on our results that:

• The presence of inspiration stridor that is intensified by efforts and shortening of aryepiglottic folds are predominant in children with laryngomalacia.
• Polysomnography is not an important parameter to indicate severity of laryngomalacia, contrarily to the presence of pectus excavatum and dissatisfactory weigh gain, which were the indicative parameters of severe laryngomalacia.
• Surgical section of aryepiglottic folds is effective with low index of morbidity.
